# Can we use MRI for timing estimation of rotator cuff tears?

**DOI:** 10.1007/s11547-026-02248-3

**Published:** 2026-06-26

**Authors:** Domenico Albano, Salvatore Gitto, Carmelo Messina, Aldo Rizzo, Riccardo Giorgino, Carlo Perfetti, Giuseppe Davide Albano, Kholoud I. A. Abdelmaguid, Saleha Irfan Arodia, Federico Ambrogi, Angelo Vanzulli, Luca Maria Sconfienza

**Affiliations:** 1 Dipartimento di Scienze Biomediche, Chirurgiche ed Odontoiatriche, Università di Milano, Via della Commenda 10, 20122 Milano, Italy; 2 Unità di Radiologia, ASST Grande Ospedale Metropolitano Niguarda, Piazza Ospedale Maggiore 3, 20162 Milano, Italy; 3 Department of Biomedical Sciences for Health, Università Degli Studi di Milano, Via Festa del Perdono, 7, 20122 Milan, Italy; 4 IRCCS Istituto Ortopedico Galeazzi, Via Cristina Belgioioso 173, 20157 Milan, Italy; 5 UOC Radiodiagnostica, ASST Centro Specialistico Ortopedico Traumatologico Gaetano Pini-CTO, P.Zza Cardinal Ferrari, 1, 20122 Milan, Italy; 6 Department of Precision and Regenerative Medicine and Ionian Area (DiMePreJ), University of Bari Aldo Moro, 70121 Bari, Italy; 7 Department of Health Promotion, Mother and Child Care, Internal Medicine and Medical Specialties, Institute of Legal Medicine, University of Palermo, Via del Vespro 129, 90100 Palermo, Italy; 8 Dipartimento di Scienze Cliniche e di Comunità, Università Degli Studi di Milano, Via Festa del Perdono, 7, 20122 Milan, Italy; 9 Laboratory of Biostatistics and Data Management, IRCCS Policlinico San Donato, Piazza Edmondo Malan, 2, 20097 San Donato Milanese, Italy; 10 Department of Oncology and Hemato-Oncology, Università Degli Studi di Milano, Via Festa del Perdono, 7, 20122 Milan, Italy

**Keywords:** Rotator cuff, Tendon, Trauma, Injury, Forensic, Magnetic resonance imaging

## Abstract

**Purpose:**

To evaluate whether routinely assessed MRI features can reliably estimate the timing of rotator cuff tears (RCTs) and to assess the performance of an MRI-based logistic regression model in distinguishing recent from chronic tears.

**Material and methods:**

In this retrospective single-center study, 255 patients with clinically and MRI-confirmed RCT following shoulder trauma underwent MRI between 2011 and 2024. Tears were classified as acute (< 6 weeks), subacute (6–12 weeks), or chronic (> 12 weeks) based on the reported date of trauma; acute and subacute tears were grouped as “recent”. Ten predefined MRI features were independently assessed. Univariable diagnostic performance metrics were calculated for each feature. A multivariable Firth-penalized logistic regression model was developed to discriminate recent from chronic tears, with internal validation performed using bootstrap resampling.

**Results:**

Among the 255 patients (mean age 58.3 years; 65% male), intra-/peri-muscular edema (42% in recent vs 12% in chronic tears) and frayed, hyperintense tendon fibers emerged as independent predictors of recent injury (odds ratios 4.15 and 6.78, respectively). However, traditional markers of chronicity—including fatty infiltration, muscle atrophy, tendon retraction, and superior humeral head migration—showed limited discriminative value. The multivariable model demonstrated modest performance, with an ideal area under the curve (AUC) of 0.74 and an optimism-corrected AUC of 0.68. Sensitivity and specificity at the optimal threshold were 56% and 82%, respectively.

**Conclusion:**

Although selected MRI findings are associated with recent RCT, an MRI-based logistic regression model provides limited accuracy for timing estimation.

## Introduction

Distinguishing the traumatic origin of rotator cuff tears (RCTs) from degenerative causes is often challenging, as tendon injuries frequently occur on a background of pre-existing degenerative changes, with trauma often acting as a contributing or precipitating factor. Another significant challenge is the dating of RCT—an area in which radiologists may be called upon to assist, particularly in the context of medico-legal evaluations related to compensation claims following injury or accidents. Such assessments are typically based on the patient’s self-reported history regarding the mechanism of trauma, along with risk factors such as age, occupational or athletic activity, clinical findings, and imaging results. Radiologists are increasingly being engaged, particularly in their role as private consultants, to provide expert evaluations for courts or insurance companies concerning musculoskeletal injuries resulting from trauma [1]. The radiologist is tasked with determining whether a causal relationship exists between the traumatic event and the RCT. A central aim of this process is to estimate the time elapsed since the injury occurred, as this helps establish a reliable timeline and clarify the correlation between trauma and the resulting pathology, particularly in cases where discrepancies arise between medical findings and the patient’s account. Furthermore, both the timing estimation and the traumatic nature of RCT may influence the clinical decision-making by the surgeon in terms of treatment indication and surgical strategy [2]. However, this evaluation is often challenging, especially considering the high prevalence of minimally symptomatic RCT. Indeed, approximately 25% of individuals in their 60 s present with full-thickness RCT, with the prevalence rising to 50% in those over 80 years of age [3], thus making it difficult to attribute a clear link between a specific traumatic event and the radiological detection of RCT.

Ultrasound is the first-line imaging modality commonly used for the evaluation of the shoulder, offering diagnostic accuracy for RCT comparable to MRI and MR arthrography [4–6]. In addition to identifying tendon tears and assessing the degree of tendon retraction, ultrasound also enables the detection of certain secondary findings potentially related to chronicity, such as glenohumeral osteoarthritis and muscle atrophy. However, its use in medico-legal settings is limited, primarily because it does not generate a standardized, permanent record of images that can be independently reviewed by a second expert for verification or second opinion. In the context of RCT, MRI plays a pivotal role, providing objective, reproducible data that can be independently reviewed by third parties and is highly informative for evaluating injury chronicity by identifying various stages of tendon damage, degeneration, and healing. Indeed, compared with ultrasound, MRI allows a more detailed—and, importantly, simultaneous—assessment of tendon morphology, muscle quality, and secondary joint alterations, which may evolve differently over time and provide complementary temporal information.

Accordingly, we conducted a retrospective analysis of patients with clinically and MRI-confirmed RCT at varying stages of lesion evolution. The aim was to identify which MRI features most significantly correlate with lesion chronicity and to develop a logistic regression model capable of estimating the age of the RCT. The goal is to provide objective accuracy data that may assist radiologists and clinicians in distinguishing recent traumatic RCT from chronic degenerative tears.

## Materials and methods

This study was approved by the Institutional Review Board of Lombardia 1, Milan, Italy (RETRORAD protocol, CE: 61/INT/2017, approved on 12th January 2026), which granted a waiver for informed consent. Following the matching of imaging, laboratory, and surgical data, the database was fully anonymized to eliminate any links between the data and patients’ identities, in compliance with the General Data Protection Regulation for Research Hospitals.

### Study protocol

This study was conducted at (IRCCS Ospedale Galeazzi-Sant’Ambrogio, Milan, Italy), in accordance with the Strengthening the Reporting of Observational Studies in Epidemiology (STROBE) declaration. A retrospective review was performed of emergency department discharge records for shoulder trauma between 2011 and 2024. The study included patients who received a clinical diagnosis or had a clinical suspicion of RCT in the emergency setting and subsequently underwent shoulder MRI at our institution within days, weeks, or months following the traumatic event, with MRI confirmation of RCT. All included patients also underwent follow-up orthopedic evaluations at our Institution after MRI. Exclusion criteria were as follows: (i) absence of MRI examination; (ii) MRI negative for RCT; (iii) non-diagnostic MRI due to motion or other imaging artifacts; (iv) unknown date of trauma; and (v) history of prior rotator cuff repair surgery or glenohumeral arthroplasty.

### MRI protocol

MRI examinations were retrospectively reviewed and acquired using various 1.5 T scanners (Siemens Avanto 1.5 T, Siemens Espree 1.5 T, GE Signa 1.5 T) and a 3.0-T scanner (GE Premier 3.0 T). Surface coils were used, and a standard field of view of 16 cm—adjusted according to patient body habitus—was applied. The imaging protocol consistently included coronal T1-weighted and fat-saturated proton density (FS-PD) sequences, sagittal T2-weighted and FS-PD sequences, and axial FS-PD sequences.

### MRI interpretation

Few imaging studies have specifically investigated the temporal classification of RCT [7]. Consequently, a set of ten imaging features was evaluated on MRI to identify direct or indirect indicators of RCT. Similar MRI studies focused on ACL injuries have proposed varying temporal classifications, typically dividing lesions into acute, subacute, intermediate, and chronic phases using different thresholds [8, 9]. In the present study, RCT were categorized as acute (within six weeks), subacute (between six and twelve weeks), and chronic (beyond twelve weeks). For further sub-analyses, acute and subacute tears were grouped together and referred to as “recent tears”.

**Fig. 1:**
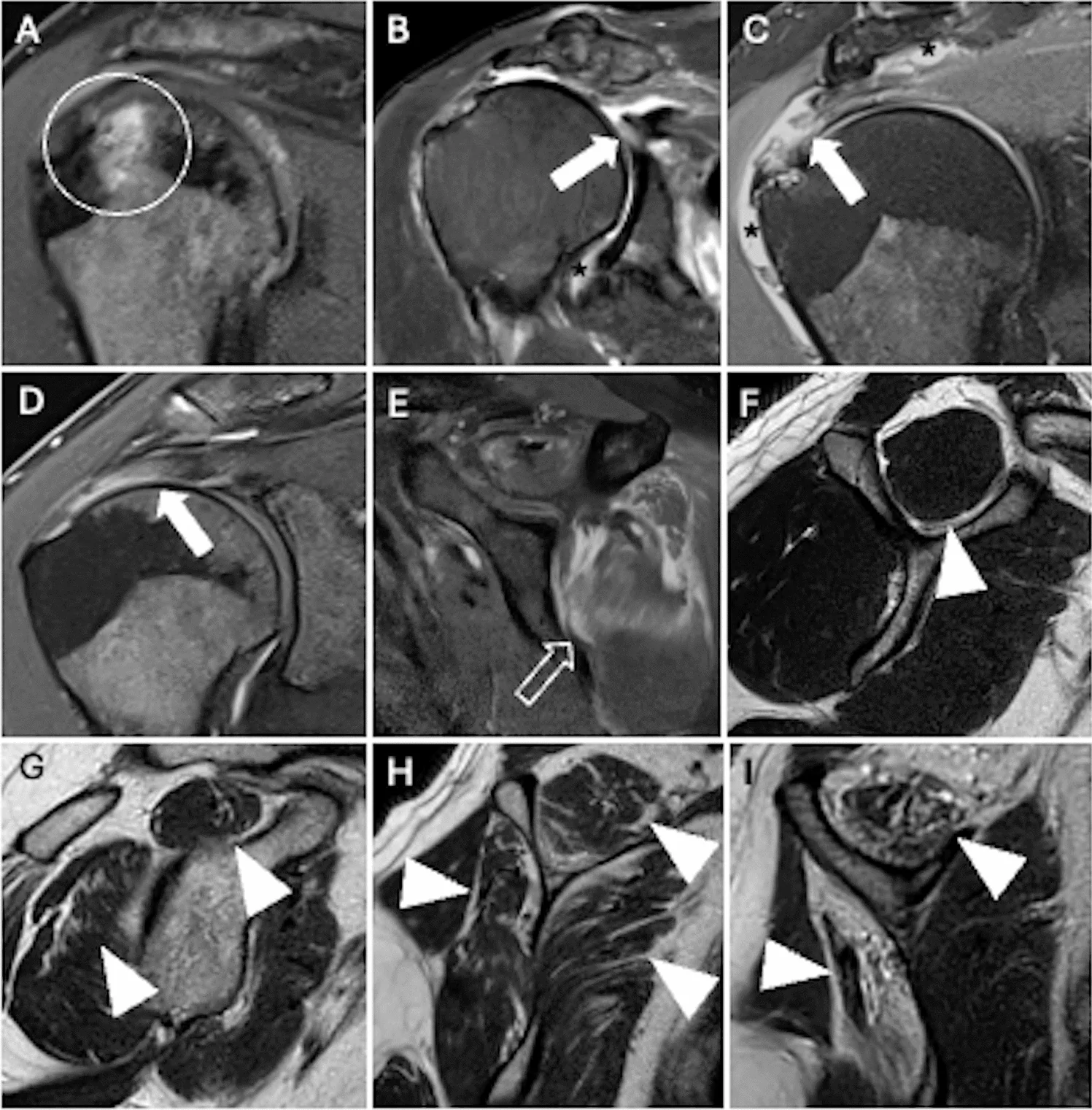
**A** Bone marrow edema (circle) of the humeral head. **B** Joint effusion (asterisk) in the axillary pouch, torn supraspinatus tendon with rounded and FS-PD hypointense fibers (arrow), which are retracted to the glenoid (Patte 3), and superior migration of the humeral head. **C** Subacromial–Subdeltoid Bursitis (asterisks) and torn supraspinatus tendon with frayed and FS-PD Hyperintense fibers (arrow) and minimal retraction (Patte 1). **D** Supraspinatus tendon tear with proximal stump (arrow) retracted to the level of the humeral head (Patte 2). **E** Intra- and peri-muscular edema of the infraspinatus muscle (void arrow). **F** Normal trophism of the rotator cuff (arrowhead) without fatty infiltration (Goutallier 0). G-H-I) Decreased trophism and different degrees of fatty infiltration of rotator muscles (arrowheads): **G** Goutallier 1 of the supraspinatus and infraspinatus; **H** Goutallier 2 of the supraspinatus, infraspinatus, and subscapularis muscles; **I** Goutallier 3 of the supraspinatus and Goutallier 4 of the infraspinatus

The imaging parameters assessed in this study included the following (Fig. 1) [1, 7, 10–12]:

- Bone edema: bone marrow edema visible on MRI as low signal on T1 and high signal on fluid-sensitive images with typical anatomic distribution.
- Joint effusion: Defined as increased intracapsular fluid with typical homogeneous signal.
- Subacromial–Subdeltoid Bursitis: Following an inflammatory and/or post-traumatic insult to the shoulder, distension of the subacromial–subdeltoid bursa may occur due to the accumulation of reactive fluid. Under normal physiological conditions, this bursa is not visible on imaging as it remains collapsed. The signal characteristics of the fluid may appear homogeneous or heterogeneous depending on the nature of its internal contents.
- Intra- and/or Peri-muscular Edema: Reactive fluid may accumulate as a result of myofibrillar injury, appearing interspersed within the muscle fibers or in the surrounding peri-muscular connective tissue.
- Frayed and T2/FS-PD Hyperintense Tendon Fibers: Torn tendon stumps often display irregular margins and increased signal intensity on T2/FS-PD sequences due to fluid infiltration. These findings are more apparent when there is a significant gap between the tendon ends.
- Rounded and T2/FS-PD Hypointense Tendon Fibers: In some cases, the torn tendon ends may present with smooth margins and retain their normal low signal intensity on T2/FS-PD sequences, suggesting the absence of acute pathological changes such as edema or hemorrhage.
- Superior Migration of the Humeral Head: In rotator cuff tears—particularly involving the supraspinatus—the stabilizing and centering function of the tendons is compromised, leading to cranial displacement of the humeral head and reduction of the subacromial space.
- Degree of Tendon Retraction (Patte Classification): The Patte classification is used to quantify the extent of proximal tendon stump retraction via topographic assessment on coronal images. It is divided into three grades: Grade I, minimal gap between proximal and distal stumps; Grade II, proximal stump retracted to the level of the humeral head; Grade III, proximal stump retracted to the glenoid.
- Muscle Bulk Assessment: The muscular bellies of the rotator cuff are anatomically confined to specific regions of the scapular surface, allowing for an assessment of muscle bulk based on their position relative to defined osseous landmarks. For example, the supraspinatus muscle resides within the Y-shaped supraspinous fossa, bordered anteriorly by the coracoid process and postero-inferiorly by the scapular spine. Muscle atrophy is suggested when the muscle belly no longer fully occupies this space according to the tangent line sign [11].
- Fatty Infiltration (Goutallier Classification): This grading system evaluates the degree of fatty degeneration of the muscle, ranging from Grade I to Grade IV, with increasing fatty replacement and corresponding reduction in muscle content.

All MRI features were independently and randomly evaluated by a board-certified radiologist with four years of experience in musculoskeletal imaging under the supervision of a senior musculoskeletal radiologist with 13 years of experience. The first 50 cases were evaluated together by the two reviewers, while, in the remaining examinations, doubtful cases were addressed through discussion and agreement among the reviewers themselves. For each conventional MRI feature, a binary response (present/absent) was recorded, except for Patte and Goutallier parameters that were associated with a grade according to the relative classification (1–3 and 0–4, respectively). In patients with concomitant multiple rotator cuff tendon lesions, when both recent and chronic findings coexisted (e.g., a supraspinatus tendon without muscle edema and an infraspinatus tendon with muscle edema, or one tendon showing rounded, T2/FS–PD hypointense tendon fibers and another showing frayed, T2/FS–PD hyperintense tendon fibers), only the findings attributable to a recent lesion were included in the analysis.

### Statistical analysis

Descriptive statistics reports mean ± SD unless otherwise specified. Group comparisons across independent continuous samples used the Kruskal–Wallis test. Comparisons across categorical variables used the Pearson’s Chi square test or the Fisher test when appropriate. The number of subjects and the statistical test used are indicated in the figure legends or in the appropriate text. The dataset initially included multiple entries per patient corresponding to injuries obtained at various dates. To avoid duplication, we retained only one entry per patient, prioritizing acute injuries over subacute, and subacute over chronic. For all imaging findings, sensitivity, specificity, accuracy, positive predictive value (PPV), and negative predictive value (NPV) were calculated. All variables were then included in the final logistic regression model.

Firth multivariable logistic regression model was used to handle some variables who were almost perfect separators. We estimated odds ratios based on the model’s regression coefficients. The model type and the number of subjects included in the analysis are indicated in the text. p-values of less than 0.05 were considered significant. Internal validation was performed using bootstrap resampling. Statistical analysis was performed using R.

## Results

Between 2011 and 2024, 255 patients, who met the inclusion criteria, underwent MRI imaging at IRCCS Ospedale Galeazzi-Sant’Ambrogio in Milan, Italy. Table 1 shows the patient characteristics. The age range of our population was broad with the youngest being 20 and the oldest being 82. The mean age of our population was 58.27, more men than women presented with these injuries, and the median days from injury was 47 days.

**Table 1:** Demographic data of our study population

Patient Characteristics				
Characteristic	Overall N = 255^*1*^	Acute N = 119^*1*^	Subacute N = 58^*1*^	Chronic N = 78^*1*^
Age (years)				
Mean ± (SD)	58.27 ± (12.03)	57.45 ± (12.86)	59.43 ± (10.75)	58.68 ± (11.66)
Range	20–82	20–82	40–80	21–77
Sex				
Male	165/255 (65%)	74/119 (62%)	39/58 (67%)	52/78 (67%)
Female	90/255 (35%)	45/119 (38%)	19/58 (33%)	26/78 (33%)
Days from trauma				
Median	47.00	14.00	60.50	166.00

SD = standard deviation

Table 2 demonstrates the proportion of patients who had imaging features depending on whether their injury was categorized as acute, subacute, or chronic.

**Table 2:** MRI features of our study population

MRI features by Injury type				
MRI features	Overall N = 255^*1*^	Acute N = 119^*1*^	Subacute N = 58^*1*^	Chronic N = 78^*1*^
Bone Marrow Edema				
Absent	219/255 (86%)	97/119 (82%)	51/58 (88%)	71/78 (91%)
Present	36/255 (14%)	22/119 (18%)	7/58 (12%)	7/78 (9.0%)
Joint Effusion				
Absent	68/255 (27%)	31/119 (26%)	17/58 (29%)	20/78 (26%)
Present	187/255 (73%)	88/119 (74%)	41/58 (71%)	58/78 (74%)
Subacromial–Subdeltoid Bursitis				
Absent	36/255 (14%)	18/119 (15%)	4/58 (6.9%)	14/78 (18%)
Present	219/255 (86%)	101/119 (85%)	54/58 (93%)	64/78 (82%)
Intra-/peri-Muscular Edema				
Absent	175/255 (69%)	69/119 (58%)	37/58 (64%)	69/78 (88%)
Present	80/255 (31%)	50/119 (42%)	21/58 (36%)	9/78 (12%)
Frayed & Hyperintense Tendon Fibers				
Absent	237/255 (93%)	113/119 (95%)	47/58 (81%)	77/78 (99%)
Present	18/255 (7.1%)	6/119 (5.0%)	11/58 (19%)	1/78 (1.3%)
Rounded & Hypointense Fibers				
Absent	115/255 (45%)	44/119 (37%)	26/58 (45%)	45/78 (58%)
Present	140/255 (55%)	75/119 (63%)	32/58 (55%)	33/78 (42%)
Patte classification of Tendon Retraction				
Stage 1	140/255 (55%)	69/119 (58%)	31/58 (53%)	40/78 (51%)
Stage 2	67/255 (26%)	33/119 (28%)	13/58 (22%)	21/78 (27%)
Stage 3	48/255 (19%)	17/119 (14%)	14/58 (24%)	17/78 (22%)
Muscular Atrophy				
Absent	138/255 (54%)	75/119 (63%)	28/58 (48%)	35/78 (45%)
Present	117/255 (46%)	44/119 (37%)	30/58 (52%)	43/78 (55%)
Goutallier Classification				
Grade 0	193/255 (76%)	98/119 (82%)	41/58 (71%)	54/78 (69%)
Grade 1	11/255 (4.3%)	4/119 (3.4%)	3/58 (5.2%)	4/78 (5.1%)
Grade 2	28/255 (11%)	11/119 (9.2%)	5/58 (8.6%)	12/78 (15%)
Grade 3	19/255 (7.5%)	4/119 (3.4%)	7/58 (12%)	8/78 (10%)
Grade 4	4/255 (1.6%)	2/119 (1.7%)	2/58 (3.4%)	0/78 (0%)
Superior Migration of Humeral Head				
Absent	186/255 (73%)	97/119 (82%)	39/58 (67%)	50/78 (64%)
Present	69/255 (27%)	22/119 (18%)	19/58 (33%)	28/78 (36%)

The performance of each MRI feature, for distinguishing recent (acute + subacute) from chronic RCT, is reported in Table 3. The variables with the best sensitivity were Subacromial-Subdeltoid bursitis, Goutallier Grade 1 & 2. While those with the highest specificity were Bone marrow edema, Intra-/peri-muscular edema, frayed/hyperintense fibers, and Goutallier Grade 4.

**Table 3:** Diagnostic performance of all MRI parameters

Test Performance Acute/Subacute vs Chronic							
Predictor	Sensitivity	Specificity	PPV	NPV	+ LR	− LR	Accuracy
Bone Marrow Edema	0.16	0.91	0.81	0.32	1.83	0.92	0.39
Joint Effusion (Absent)	0.26	0.73	0.29	0.69	1.06	0.98	0.58
Subacromial–Subdeltoid Bursitis	0.88	0.18	0.71	0.39	1.07	0.69	0.66
Intra-/peri-Muscular Edema	0.40	0.88	0.89	0.39	3.48	0.68	0.55
Frayed and Hyperintense Tendon Fibers	0.10	0.99	0.94	0.32	7.49	0.92	0.37
Rounded and Hypointense Fibers	0.60	0.58	0.76	0.39	1.43	0.69	0.60
Goutallier Grade 0	0.79	0.31	0.72	0.39	1.13	0.70	0.64
Goutallier Grade 1 (Absent)	0.95	0.04	0.30	0.64	1.01	0.77	0.32
Goutallier Grade 2 (Absent)	0.85	0.09	0.29	0.57	1.07	0.59	0.32
Goutallier Grade 3 (Absent)	0.90	0.06	0.30	0.58	1.05	0.61	0.32
Goutallier Grade 4	0.02	1.00	1.00	0.31	Inf	0.98	0.32
Muscular atrophy (Absent)	0.45	0.42	0.25	0.63	1.30	0.76	0.43
Patte Stage 1	0.56	0.49	0.71	0.33	1.10	0.89	0.54
Patte Stage 2 (Absent)	0.73	0.26	0.30	0.69	1.01	0.97	0.40
Patte Stage 3 (Absent)	0.78	0.18	0.29	0.65	1.05	0.80	0.36
Superior Migration of Humeral Head (Absent)	0.64	0.23	0.27	0.59	1.20	0.65	0.36

PPV = positive predictive value, NPV = negative predictive value, + LR = positive likelihood ratio, –LR = negative likelihood ratio

**Fig. 2:**
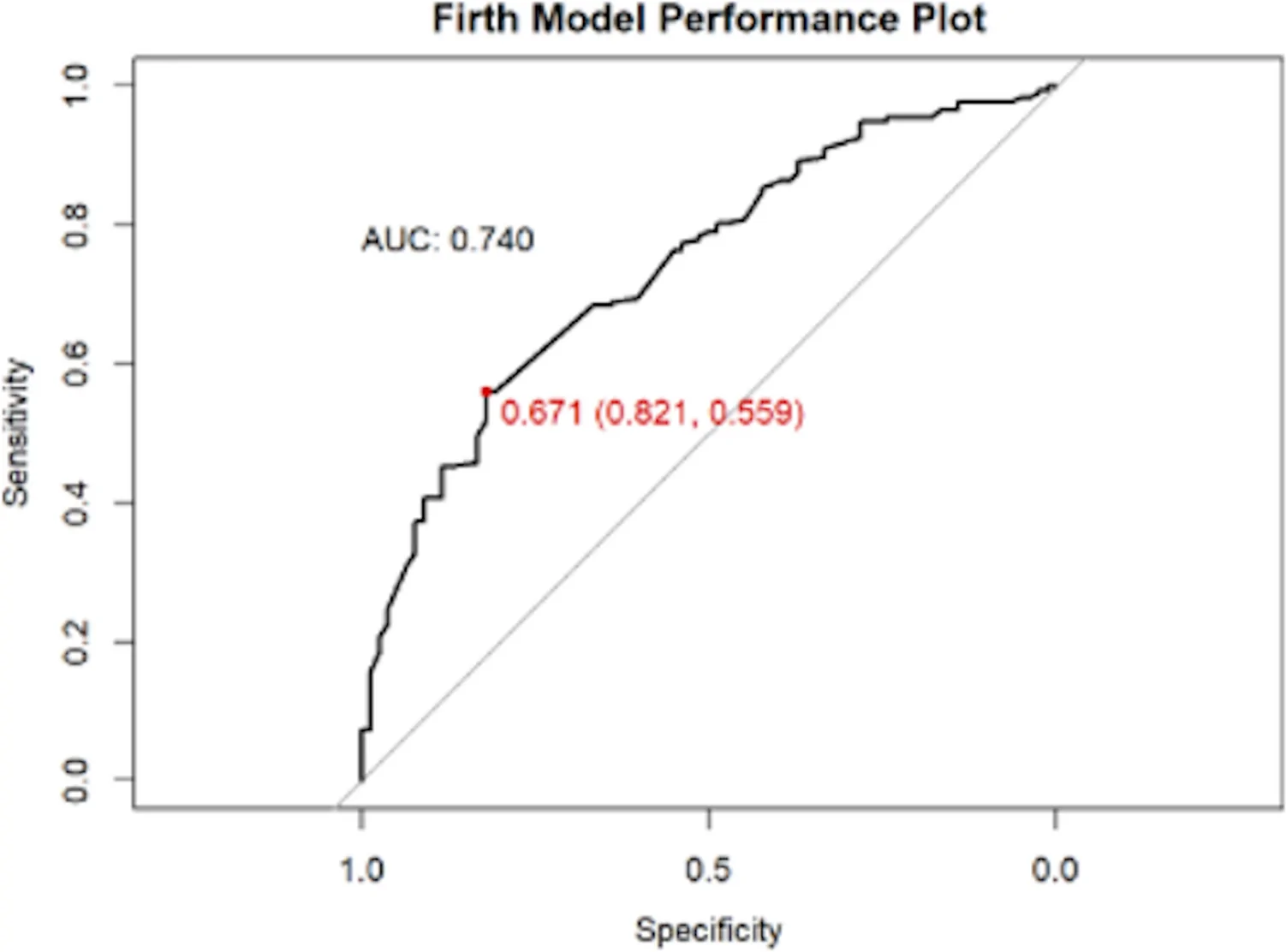
ROC curve of the Firth-penalized logistic regression model

Table 4 illustrates the results of the Firth regression model. The two significant variables were intra-/peri-muscular edema and frayed/hyperintense tendon fibers. The performance of the model (Table 4) was subpar with an ideal AUC of 0.74. The best threshold via Youden’s index was calculated to be 0.67, but the sensitivity at the threshold was 56%, with a specificity of 82%. To assess generalizability, the optimism bootstrap-corrected AUC was calculated to be 0.68. The AUC curve is reported in Fig. 2. The model and its coefficients were used to create an app that can be reached by using the following link: http://w4esst-saleha-arodia.shinyapps.io/RotatorcuffdatingusingMRI.

**Table 4:** The fit of a Firth logistic regression model for predicting the timing of RCT

Firth model Summary Recent (Acute + Subacute) vs Chronic lesions			
Characteristic	exp (Beta)	95% CI	*p*-value
Bone Marrow Edema			
Absent	–	–	
Present	1.32	0.54, 3.53	0.6
Joint Effusion			
Absent	–	–	
Present	0.89	0.43, 1.82	0.8
Subacromial–Subdeltoid Bursitis			
Absent	–	–	
Present	1.10	0.49, 2.42	0.8
Intra-/peri-Muscular Edema			
Absent	–	–	
Present	4.15	1.89, 9.95	< 0.001
Frayed Hyperintense Tendon Fibers			
Absent	–	–	
Present	6.78	1.48, 65.5	0.011
Rounded Hypointense Fibers			
Absent	–	–	
Present	1.66	0.80, 3.48	0.2
Goutallier Classification			
Grade 0	–	–	
Grade 1	0.44	0.10, 1.98	0.3
Grade 2	0.43	0.15, 1.17	0.10
Grade 3	0.46	0.15, 1.45	0.2
Grade 4	1.05	0.09, 149	> 0.9
Muscular Atrophy			
Absent	–	–	
Present	1.44	0.58, 3.73	0.4
Patte classification of Tendon Retraction			
Stage 1	–	–	
Stage 2	1.31	0.57, 3.12	0.5
Stage 3	1.58	0.58, 4.43	0.4
Superior Migration of Humeral Head			
Absent	–	–	
Present	0.57	0.25, 1.31	0.2

Performance of Firth model	
Characteristic	Value
Ideal AUC	0.74
Bias corrected AUC	0.68
Best Threshold via Youden	0.67
Sensitivity at Threshold	0.56
Specificity at Threshold	0.82
PPV at Threshold	0.88
+ LR at Threshold	3.11
-LR at Threshold	0.54

CI = Confidence interval, AUC = area under the curve, PPV = positive predictive value, + LR = positive likelihood ratio, –LR = negative likelihood ratio

## Discussion

The ability to estimate the timing of RCT based on MRI findings represents a recognized challenge in musculoskeletal radiology, particularly in medico-legal and compensation-related settings, where radiologists are increasingly asked to provide expert opinions on the causal relationship between trauma and tendon injury. Unlike purely diagnostic tasks, temporal assessment requires imaging biomarkers that evolve in a predictable and sufficiently discriminative manner over time. The present study sought to evaluate whether routinely assessed MRI features could be combined into a logistic regression model capable of distinguishing recent from chronic RCT. Our results demonstrate that, although certain MRI findings show statistically significant associations with injury chronicity, the overall predictive performance of an MRI-based model remains limited. Indeed, in the context of rotator cuff tear pathology, the biological complexity of the disease may be predominant over the temporal resolution provided by conventional morphological imaging [13].

At the univariable level, several MRI features demonstrated partial discriminatory ability. Intra- and peri-muscular edema and frayed, hyperintense tendon fibers were more frequently observed in recent tears and emerged as the only independent predictors retained in the multivariable model. These findings are biologically plausible, as muscle edema reflects acute myofibrillar injury, while frayed, hyperintense tendon margins likely correspond to recent fiber disruption with interposed fluid and hemorrhage. Conversely, features traditionally associated with chronicity—such as fatty infiltration, muscle atrophy, tendon retraction, and superior humeral head migration—showed limited specificity for chronic tears when evaluated in isolation. This aspect supports the concept that chronic findings on magnetic resonance imaging may reflect pre-existing degenerative changes rather than necessarily corresponding to true temporal markers of the index injury.

Despite these associations, the overall performance of the multivariable model was modest. The optimism-corrected AUC of 0.68, together with a sensitivity of only 56% at the optimal threshold, indicates limited reliability for clinical or medico-legal decision-making. This contrasts with more favorable results reported for temporal estimation in other musculoskeletal contexts, such as anterior cruciate ligament injuries, where clearer injury mechanisms and more distinct temporal imaging patterns are present [8, 9]. The relatively weak performance observed in our study likely reflects fundamental differences in the pathophysiology of RCT, which often arise on a background of chronic tendon degeneration, with trauma acting as a triggering or aggravating event rather than a discrete cause.

The limited discriminative power of MRI in dating RCT highlights an important conceptual issue: Many imaging features traditionally interpreted as markers of chronicity may precede the traumatic event and therefore do not reliably reflect injury age, potentially due to previous unrecognized or asymptomatic RCT. Indeed, fatty infiltration, muscle atrophy, tendon retraction, and superior migration of humeral head may be present in asymptomatic individuals or develop gradually over years, blurring the distinction between acute and chronic lesions [14]. Similarly, inflammatory findings such as bursitis and joint effusion, although common in acute settings, were highly prevalent across all injury stages and demonstrated low specificity. These observations underscore the difficulty of deriving robust temporal information from static imaging features in a condition characterized by overlapping degenerative and traumatic processes.

From a methodological standpoint, the use of Firth-penalized regression allowed for stable coefficient estimation despite sparse data and quasi-complete separation in some predictors. Internal validation with bootstrap resampling confirmed limited generalizability, with a noticeable drop in AUC after optimism correction. Importantly, this rigorous statistical approach strengthens the credibility of the negative findings and supports the conclusion that model weakness reflects intrinsic limitations of the imaging biomarkers rather than analytical shortcomings.

Clinically and medico-legally, these results have relevant implications. While MRI remains indispensable for diagnosing RCT and characterizing tendon morphology, its role in reliably estimating injury timing appears constrained. The findings of this study caution against overreliance on MRI alone for determining the traumatic origin or temporal proximity of RCT, particularly in compensation and litigation settings. Imaging findings should instead be interpreted in conjunction with clinical history, physical examination, surgical findings, and—when available—prior imaging. The modest performance of the proposed model suggests that MRI-based temporal estimation should be framed probabilistically and communicated with appropriate uncertainty.

In the study by Loew et al. on 50 patients with RCT [7], certain criteria such as muscle edema and fluid collections demonstrated moderate discriminative ability in separating acute from chronic RCT, with inflammatory signs observed in a substantially higher proportion of acute tears compared with chronic ones (e.g., increased signal intensity patterns and peritendinous changes reported in > 70% of acute cases). In contrast, in our cohort bone marrow edema was present in only 18% of acute and 9% of chronic tears, and intra-/peri-muscular edema occurred in 42% of acute versus 12% of chronic lesions, yielding a modest odds ratio of 4.15 (95% CI 1.89–9.95, *p* < 0.001) in multivariable analysis. This disparity suggests that demographic differences, underlying tendon degeneration, and variability in injury mechanisms likely attenuate the specificity of such reactive signs in a broader clinical population. Similarly, Ilyas et al. [15] reported strong correlations between symptom duration and both tendon retraction and muscle atrophy grades in young patients with isolated full-thickness supraspinatus tears. In our more heterogeneous cohort, however, neither Patte retraction stage nor muscle atrophy achieved significance in the regression model, and features of chronicity such as fatty infiltration showed a limited predictive value (all *p* > 0.2). This divergence may stem from the more uniform injury patterns and age range in Ilyas et al., compared with the broad age distribution and mixed degenerative background in our series. Collectively, these comparisons underscore that MRI features associated with RCT are influenced by type of the lesions and chronic degenerative processes common in older adults, which diminish their utility as stand-alone temporal biomarkers and likely contributes to the modest discriminative performance of our model.

Some limitations of this study must be pointed out. Its retrospective, single-center design and the inherent heterogeneity of MRI scanners and acquisition protocols may have influenced feature assessment, although this variability mirrors real-world practice. The categorization of injury timing relied on reported trauma dates, which may be subject to recall bias. Additionally, clinical variables were intentionally excluded to isolate the contribution of imaging features, but future integrative models combining MRI, clinical examination, and functional data may achieve improved performance. Then, patients were not stratified by age, which may have limited the discriminative value of some parameters that could be more informative within specific age groups [16]. Furthermore, when multiple RCT coexisted, with both recent and chronic MRI features, only the “recent” findings were considered in our analysis. This approach was necessary because, in rotator cuff pathology, multiple tendon lesions with different temporal characteristics may be present simultaneously, also in patients without history of other traumas. Indeed, RCT may be degenerative, associated with age and working/sports activities, occurring also without and trauma [17]. Last, a nonnegligible limit is the high frequency of pre-existing, degenerative, asymptomatic RCT, which acts as a confounding variable. Indeed, chronic atraumatic tears often remain asymptomatic until imaging is performed due to trauma. Hence, there is a significant risk that chronic RCTs are misclassified as acute or subacute based patient’s clinical history. This may limit the model’s predictive accuracy, making it challenging to understand the radiological timeline of RCT and to differentiate recent traumatic lesion from previous chronic tears.

In conclusion, this study demonstrates that, although certain MRI findings are associated with the recent occurrence of RCT, an MRI-based logistic regression model provides only limited accuracy for injury timing estimation. These results highlight the intrinsic complexity of RCT pathophysiology and the substantial overlap between traumatic and degenerative imaging features. MRI should therefore be regarded as a supportive rather than definitive tool for temporal assessment of RCT, and its findings should be integrated within a comprehensive clinical and medico-legal framework. Future research should focus on multimodal approaches and prospective validation to better address this unmet clinical and forensic need.
